# Effect of the Tetravalent Dengue Vaccine TAK-003 on Sequential Episodes of Symptomatic Dengue

**DOI:** 10.4269/ajtmh.22-0673

**Published:** 2023-03-06

**Authors:** Xavier Sáez-Llorens, Shibadas Biswal, Charissa Borja-Tabora, LakKumar Fernando, Mengya Liu, Derek Wallace, Nicolas Folschweiller, Humberto Reynales, Inge LeFevre, for the TIDES Study Group

**Affiliations:** ^1^ Hospital del Niño Dr. José Renán Esquivel, Sistema Nacional de Investigación at SENACYT, Centro de Vacunación Internacional (Cevaxin), Panama City, Panama;; ^2^ Takeda Vaccines, Inc., Cambridge, Massachusetts;; ^3^ Research Institute For Tropical Medicine, Muntinlupa, Philippines;; ^4^ Centre for Clinical Management of Dengue & Dengue Haemorrhagic Fever, Negombo General Hospital, Negombo, Sri Lanka;; ^5^ Takeda Pharmaceuticals International AG, Zurich, Switzerland;; ^6^ Centro de Atención e Investigación Médica, CAIMED, Bogotá, Colombia

## Abstract

In the pivotal phase 3 efficacy trial (NCT02747927) of the TAK-003 dengue vaccine, 5 of 13,380 TAK-003 recipients and 13 of 6,687 placebo recipients experienced two episodes of symptomatic dengue between the first dose and the end of the study, ∼57 months later (patients received the second dose 3 months after the first dose). Two of these participants experienced repeat infection with the same serotype (i.e., homotypic reinfection). In comparison with placebo, the relative risk of a subsequent episode of symptomatic dengue was 0.19 (95% CI, 0.07–0.54) in TAK-003 recipients. Based on the small number of subsequent episodes, these data suggest a potential incremental effect of TAK-003 beyond prevention of the first episode of symptomatic dengue after vaccination.

## INTRODUCTION

Dengue is a mosquito-borne viral disease that is endemic in more than 100 countries worldwide, predominantly in tropical and subtropical regions.^1^ Infection by one of the four dengue virus (DENV) serotypes is thought to confer lifelong homotypic immunity, but may also confer temporary cross-protection against the heterotypic serotypes.^2^^,^^3^ Hence, study of sequential serotype-confirmed dengue infections in the same individual requires meticulous data collection in long-term or cohort studies. The clinical outcome of subsequent infections is believed to be influenced by many factors, such as the number of reinfections, sequence of serotypes, and time interval between the infections, in addition to the well-known risk of severe disease manifestation upon secondary infection.^3^^–^^6^

The tetravalent, recombinant, live-attenuated dengue vaccine (TAK-003), which is based on a DENV serotype 2 (DENV-2) backbone, is currently being assessed in a large-scale, long-term, phase 3 efficacy trial in healthy children and adolescents living in dengue-endemic areas (NCT02747927).^7^^–^^9^ The surveillance of febrile illnesses in this trial was designed to detect all symptomatic cases (both nonhospitalized and hospitalized dengue) throughout the trial, thus providing a unique opportunity for identifying symptomatic sequential infections. We have reported previously^10^ that TAK-003 is efficacious against symptomatic dengue both in baseline seronegative and seropositive participants, with a profile of variable performance against individual serotypes. Efficacy was demonstrated against all four serotypes in the baseline seropositive subpopulation, and against DENV-1 and -2 in the baseline seronegative subpopulation. In the latter subpopulation, the available data did not suggest efficacy against DENV-3, and the case counts were too small to assess efficacy against DENV-4.

During the trial, vaccine efficacy was estimated using a Cox proportional hazards model, and only the first episodes of virologically confirmed dengue (VCD) were considered in the efficacy estimation. For serotype-specific efficacy, again only the first episodes caused by that specific serotype were considered. Because of the long duration of active febrile surveillance, we were able to document participants who experienced multiple VCD episodes from the first dose until 4.5 years after the two doses of trial vaccination. Herein, we describe these multiple episodes of symptomatic dengue, together with an exploratory estimate of the effect of vaccination on subsequent dengue episodes.

## METHODS

The dengue episodes reported in this article were observed during a phase 3 randomized, double-blind, placebo-controlled trial of TAK-003 in eight countries in Latin America and Asia considered to be endemic for dengue (NCT02747927). During the trial, healthy children and adolescents age 4 to 16 years were randomized 2:1 to receive either two doses of TAK-003 (months 0 and 3) or placebo. Full details of enrollment criteria and trial procedures have been published previously.^7^^–^^9^ The trial was conducted in accordance with the Declaration of Helsinki and the principles of Good Clinical Practice, and informed assent/consent was obtained from participants and their parents or legal guardians prior to enrollment.

In brief, the multipart trial had up to 4.5 years of follow-up for individual participants after administration of the two doses of TAK-003 or placebo,^7^^–^^9^ and has another ongoing 25 months for those age 4 to 11 years at randomization who enrolled to participate in a follow-up booster evaluation phase. Febrile surveillance during all parts of the trial includes at least weekly contact with participants or their legal guardians for robust detection of all symptomatic dengue cases using serotype-specific reverse transcription–polymerase chain reaction (RT-PCR) testing in the acute samples. Virologically confirmed dengue was defined as a febrile illness (body temperature ≥ 38 °C for 2 of any 3 consecutive days) or illness clinically suspected to be dengue by the investigator in association with a positive serotype-specific RT-PCR result. Severity of hospitalized dengue cases was assessed by an adjudication committee (severity criteria reported previously^7^^–^^9^) and by a program that analyzed data to identify VCD cases meeting WHO 1997 dengue hemorrhagic fever criteria.^11^

Dengue RNA was detected and quantified with a validated serotype-specific RT-PCR assay. The upper limit of quantification (ULoQ) was determined to be 85,714,286 genome copy equivalents per milliliter (log_10_ [ULoQ] = 7.9) for all four DENV serotypes. Serostatus, based on the presence or absence of DENV neutralizing antibodies determined using a microneutralization test, was assessed at baseline for all participants. Microneutralization test results are expressed as the reciprocal of the dilutions of test serum that show a 50% reduction in plaque counts compared to the virus controls. Seropositivity was defined as a neutralizing antibody titer of ≥ 10 to at least one DENV serotype.

Descriptive details of VCD in participants who experienced multiple episodes between the first dose and 4.5 years after the second dose are presented. Relative risk was calculated as the number of events divided by the number of participants in the TAK-003 group, over the number of events divided by the number of participants in the placebo group. All the analyses presented in this manuscript are exploratory and post hoc in nature.

## RESULTS

In total, 13,380 participants in the TAK-003 group and 6,687 in the placebo group received at least one dose of TAK-003 or placebo between September 2016 and March 2017, and were included in the safety population by vaccination group. There were an additional four participants who received both TAK-003 and placebo as a result of an administrative error. Of the 20,063 participants who were tested at baseline, 5,547 (27.6%) were seronegative to all four serotypes.^8^

In ∼57 months after the first dose, 442 participants (3.3%) in the TAK-003 group and 547 (8.2%) in the placebo group experienced at least one VCD episode (Table 1). No coinfections of multiple serotypes were noted. Five participants (0.04%) in the TAK-003 group and 13 (0.19%) in the placebo group experienced two VCD episodes during this time frame, resulting in a relative risk of the subsequent VCD episode versus placebo of 0.19 (95% CI, 0.07–0.54) based on the safety set, and 0.48 (95% CI, 0.17–1.32) based on the population of participants who had experienced at least one VCD episode. Although the latter analysis involved a postrandomization subset population and a nonstatistically conclusive estimate, both are directionally similar and suggest a favorable effect.

**Table 1:** Number of participants and relative risk of a subsequent episode of virologically confirmed dengue (VCD) from the first dose to 4.5 years after the second dose (approximately month 57), safety set data

Variable	TAK-003 (*n* = 13,380)	Placebo (*n* = 6,687)
Participants with ≥ 1 episode of VCD, *n* (%)	442 (3.3)	547 (8.2)
Participants with subsequent VCD episode, *n* (%)	5 (< 0.1)	13 (0.2)
Relative risk of subsequent VCD episode* (95% CI)	0.19 (0.07–0.54)	
Relative risk of subsequent VCD episode in participants with ≥ 1 VCD episode (95% CI)	0.48 (0.17–1.32)	

VCD = virologically confirmed dengue.

* Based on total safety set.

Ten of the sequential episodes of VCD occurred at the sites in the Philippines, four in Colombia, three in Sri Lanka, and one in Thailand (Table 2). Ten of these 18 participants were seropositive at baseline. All but two of the participants who experienced two episodes during the trial were children age 4 to 8 years at the time of randomization. Subsequent episodes occurred 46 to 1,181 days after the first episode recorded in the trial (mean, 500 days). Of the subsequent episodes, five were DENV-1, seven were DENV-2, three were DENV-3, and three were DENV-4. Details of the treatment group are not provided to prevent unblinding in the ongoing study. There were two instances of homotypic VCD: one DENV-3 case occurring 763 days after the first episode in a male participant from the Philippines who was 12 years old at enrollment and seropositive at baseline, and one DENV-1 case occurring 207 days after the first episode in a 7-year-old seronegative female participant from Colombia.

**Table 2:** Clinical and laboratory data of participants who experienced two episodes of virologically confirmed dengue infection during the trial from the first dose to 4.5 years after the second dose (approximately month 57), safety set data

Country	Gender	Age*, years	Baseline neutralization titer				First episode recorded during the trial						Subsequent episode recorded during the trial					
			DENV-1	DENV-2	DENV-3	DENV-4	Onset after dose 1, days	Serotype	Clinical dengue	Duration of fever, days	Genome copy equivalent/mL	Hospitalized	Onset after previous episode, days	Serotype	Clinical dengue	Duration of fever, days	Genome copy equivalent/mL	Hospitalized
Baseline seropositive																		
Colombia	F	12	28	27	< 10	< 10	1,082	DENV-1	N	6	27,633,100	N	540	DENV-2	N	6	> ULoQ	N
Colombia	M	4	< 10	< 10	460	< 10	875	DENV-1	Y	5	19,367,543	N	831	DENV-2	N	3	5,202,452	N
Philippines	F	4	151	798	563	2,685	572	DENV-1	N	5	> ULoQ	N	506	DENV-3	N	5	6,627	N
Philippines	M	6	88	4,432	63	58	339	DENV-1	N	4	> ULoQ	N	1,181	DENV-4	N	3	1,940,672	N
Philippines	M	5	< 10	< 10	< 10	231	948	DENV-2	N	4	> ULoQ	N	114	DENV-1	N	7	1,505,325	N
Sri Lanka	F	6	14	25	21	110	345	DENV-2	Y	5	> ULoQ	Y	700	DENV-1	N	4	3,916,150	N
Philippines	M	6	19	25	< 10	554	6	DENV-2	N	6	> ULoQ	N	46	DENV-3	N	5	3,348,823	N
Philippines	F	8	34	13	< 10	< 10	577	DENV-3	N	3	35,468,967	N	309	DENV-2	N	4	> ULoQ	N
Philippines	M	12	105	4,374	146	330	297	DENV-3	N	3	25,121,129	N	763	DENV-3	N	3	135,264	N
Philippines	F	5	< 10	12	17	< 10	237	DENV-4	N	4	7,529,383	N	774	DENV-2	N	4	> ULoQ	N
Baseline seronegative																		
Colombia	F	7	< 10	< 10	< 10	< 10	1,001	DENV-1	N	3	15,556	N	207	DENV-1	Y	6	> ULoQ	Y
Philippines	M	7	< 10	< 10	< 10	< 10	948	DENV-1	N	5	28,182,451	N	753	DENV-2	N	4	> ULoQ	N
Sri Lanka	F	8	< 10	< 10	< 10	< 10	298	DENV-1	Y	5	124,166	Y	191	DENV-2	N	3	1,389,205	N
Colombia	M	8	< 10	< 10	< 10	< 10	1,142	DENV-2	N	5	224,670	N	375	DENV-1	N	4	2,135	N
Sri Lanka	M	7	< 10	< 10	< 10	< 10	564	DENV-2	N	3	73,814	N	502	DENV-1	N	2	316,632	N
Thailand	M	4	< 10	< 10	< 10	< 10	1,134	DENV-2	Y	4	2,847,520	N	364	DENV-4	Y	5	2,518,086	Y
Philippines	M	6	< 10	< 10	< 10	< 10	706	DENV-3	N	2	15,506,896	N	310	DENV-2	N	2	> ULoQ	N
Philippines	F	8	< 10	< 10	< 10	< 10	426	DENV-3	Y	5	> ULoQ	Y†	533	DENV-4	N	4	6,809,951	N

DENV = dengue virus; F = female; M = male; N = no; ULoQ = upper limit of quantification; Y = yes.

* Age at randomization.

† Classified as severe dengue by the adjudication committee.

Treatment group is not provided to prevent participant-level unblinding because the trial is ongoing and remains blinded. Dengue RNA was detected and quantified with a validated dengue detection reverse transcription–polymerase chain reaction. The ULoQ of the method was determined to be 85,714,286 genome copy equivalents for all four dengue serotypes. Microneutralization test results are expressed as the reciprocal of the dilutions of test serum that show a 50% reduction in plaque counts compared to the virus controls.

In the 18 participants who experienced two VCD episodes, five of the first episodes after trial vaccination were diagnosed clinically as dengue by the investigators, and three participants were hospitalized (one episode each of DENV-1, -2, and -3). Two of the subsequent episodes (DENV-1 homotypic reinfection and DENV-4 following DENV-2) were diagnosed clinically as dengue and both required hospitalization. One of the first VCD episodes (DENV-3 in a baseline seronegative participant) was classified as severe by the adjudication committee; none of the other episodes were classified as severe or dengue hemorrhagic fever.

## DISCUSSION

Active febrile surveillance over ∼57 months in this phase 3 efficacy trial enabled the identification of 18 multiple, symptomatic dengue infections. Although this is a small number of cases, the distribution by vaccination group (five in the TAK-003 group versus 13 in the placebo group; 2:1 randomization ratio) provides some evidence of the lower risk of a subsequent symptomatic dengue episode in people who have postvaccination breakthrough cases. This incremental effect of TAK-003 is relevant because people living in dengue-endemic countries are at risk of multiple, sequential dengue infections during their lifetime.

In earlier reports from this ongoing trial, we noted a trend of a lower proportion of breakthrough cases in vaccinees presenting with dengue-relevant clinical characteristics compared with the placebo group, such as signs of plasma leakage, thrombocytopenia, or signs of bleeding.^9^ These data, together with the data reported herein, suggest that the immune response to TAK-003 may have an attenuating effect on the clinical manifestation of dengue infections. It is plausible to hypothesize that TAK-003 vaccination and subsequent breakthrough infections might help in transitioning to a postsecondary-like state so that subsequent infections are less symptomatic. In this context, the breakthrough infections (both symptomatic and asymptomatic) in vaccinated individuals can potentially serve as natural boosters. Younger participants (4–11 years at enrollment), who made up the majority of these sequential cases, are now being evaluated further after the administration of a booster dose in the ongoing trial.

Among the 18 sequential episodes, the majority of the cases (i.e., 13 of 18 first episodes and 12 of 18 subsequent episodes) were caused by DENV-1 or -2. This reflects the data in the placebo group over ∼57 months in eight dengue-endemic countries, in which these two serotypes accounted for the majority of dengue cases (423 of 560). This observed serotype distribution pattern also aligns generally with decades of dengue epidemiology globally.^12^

Notably, we observed no clear patterns in causative serotype of sequential episodes, but we did record two cases of homotypic reinfection. The first case was a DENV-3 infection in a baseline seropositive participant who most likely had at least one DENV-2 infection prior to enrollment in the trial, based on the baseline neutralizing titers (DENV-1, 105; DENV-2, 4,374; DENV-3, 146; and DENV-4, 330), although this was not confirmed. This observation is particularly interesting because TAK-003 vaccination did not show efficacy against DENV-3 in baseline seronegative participants.^7^^,^^9^ Most DENV-3 cases in the trial, including the homotypic VCD reinfection, were reported at the sites in the Philippines. The second case was a DENV-1 reinfection in a baseline seronegative participant who was hospitalized for the subsequent episode but not the first. Although it is believed that dengue infections provide complete and lifelong protection against the same serotype, recent findings have questioned this existing dogma.^13^ It may be, in part, because the cases are difficult to detect outside the settings, with a long duration of follow-up, as well as laboratory testing of all febrile illnesses with serotype-specific PCR. The potential for homotypic reinfection poses additional complexities in dengue vaccine development.

The majority of dengue infections tend to be asymptomatic^14^; hence, it is likely there were many more subsequent asymptomatic infections than the 18 symptomatic sequential cases identified in the trial. These asymptomatic cases might have also altered the immunological profile of the trial population to some extent. However, we believe the placebo control minimizes any potential bias on our conclusions. In addition, the few symptomatic sequential cases in the TAK-003 group did not allow for robust comparison of symptoms between the earlier and the later episodes. These are some of the limitations in this exploratory analysis besides the overall small number of cases.

## CONCLUSION

In conclusion, the available data suggest that TAK-003 vaccination resulted in a reduced risk of experiencing sequential episodes of symptomatic dengue in children and adolescents age 4 to 16 years in dengue-endemic areas. These data indicate some potential benefit even in vaccine recipients who might experience breakthrough symptomatic dengue.
